# Rapid Detection of Flusilazole in Pears with Au@Ag Nanoparticles for Surface-Enhanced Raman Scattering

**DOI:** 10.3390/nano8020094

**Published:** 2018-02-08

**Authors:** Zhihui Zhao, Yiqun Huang, Yuxia Fan, Keqiang Lai

**Affiliations:** 1 College of Food Science and Technology, Shanghai Ocean University, No. 999 Hucheng Huan Road, Lin Gang New City, Shanghai 201306, China; zhihuiz2016@163.com (Z.Z.); yiqunh@csust.edu.cn (Y.H.); yxfan@shou.edu.cn (Y.F.); 2 School of Chemistry and Biological Engineering, Changsha University of Science & Technology, Changsha 410004, China; 3 Engineering Research Center of Food Thermal Processing Technology, Shanghai Ocean University, Shanghai 201306, China

**Keywords:** surface-enhanced Raman spectroscopy, pesticide, pear, flusilazole, Au@Ag

## Abstract

Residual pesticides in vegetables or fruits have been become one of the world’s most concerned food safety issues. Au-Ag core-shell nanoparticles (Au@Ag NPs) coupled with surface-enhanced Raman spectroscopy (SERS) was used for analysis of flusilazole which was widely applied in pears. Three different diameters of Au@Ag NPs were prepared to select the best SERS substrate for analyzing flusilazole. The Au@Ag NPs sizes of 90 ± 7 nm showed the highest enhancement effect and could be detected flusilazole standard solution and the minimum detectable concentration was 0.1 mg/L. Flusilazole in pear could also identified at as low as 0.1 μg/g. The amount of adsorbent is critical in the sample preparation process and the best amount of each absorber dosage was 0.6 g MgSO_4_, 0.2 g C_18_ and 0.2 g primary secondary amine (PSA). The experimental results indicated a good linear relationship between the Raman intensities of chief peaks and the concentrations of flusilazole solutions (*R*^2^ = 0.924–0.962). This study shows that Au@Ag as SERS substrate has great potential to analyze of flusilazole in food matrices.

## 1. Introduction

Flusilazole, 1-[[bis(4-fluorophenyl)methylsilyl]methyl]-1H-1,2,4-triazole is a highly effective and broad-spectrum organosilicon fungicide that has been widely used to control plant diseases such as black spot or scab of pear, powdery mildew on cereal, wheat leaf rust and stripe rust and barley leaf spot [1]. To ensure the safety of food supplies, the maximum residual levels of flusilazole in certain plant source foods are regulated, which generally ranged from 0.05 μg/g (e.g., soybean, beetroot) to 0.5 μg/g (e.g., melons, small berries) depending on the types of foods [2]. Similar to the analysis of other residual pesticides, quantification of flusilazole in plant source foods is mainly based upon chromatographic methods such as high-performance liquid chromatography (HPLC), liquid chromatography-tandem mass spectrometry (LC-MS/MS), gas chromatography–tandem mass spectrometry (GC-MS/MS) [3,4,5]. However, chromatography-based analytical methods are usually expensive, lengthy and require professional operation. It has been always of significance to develop a simplified method to replace the chromatographic based methods. During the past decade, surface-enhanced Raman spectroscopy has attracted increasing concern from researchers in the area of analyzing trace chemical hazards in various foods, agricultural produce and environments [6,7,8,9,10,11,12,13]. Among all the potential applications of SERS, detection of residual insecticides, herbicides and fungicides in fruits and vegetables is one of the most active areas. For examples, Zhu synthesized multi-branched gold nanostars as SERS substrate for analysis of thiram and the lowest detected was 10^−10^ M in solution and 0.24 ng/cm^2^ in apple peels [14]; Li reported a simple method to prepare core-shell Ag_2_O@Ag NPs with Polymethyl methacrylate (PMMA) for the detection of chlorpyrifos with as low as 10^−7^ M on apple and cucumber peels [15]; Fang used Ag NPs as flexible SERS substrate to detect 10^−9^ M paraquat on the apple and pear peels [16]; Luo fabricated Au NPs to detect phosmet and thiabendazole in apple and the lowest detectable level was 0.5, 0.1 μg/g, respectively [17].

Some special substrates, mainly noble metals (e.g., Au and Ag) with nanoscale roughened surfaces or colloidal nanoparticles, have to be used to achieve tremendous enhancement effect for the Raman signal of a trace chemical. However, due to the complex nature of SERS phenomena, there is no universal substrate that can be applied for detection of all different chemicals and choosing an appropriate substrate is generally considered as the most important factor to achieve successful SERS applications [18,19]. Silver and gold bimetallic nanoparticles have shown tremendous advantages over commonly used gold or silver substrates [20]. Au-Ag core-shell nanoparticles (Au@Ag) could overcome the instability of silver NPs while keep the advantage of its higher enhancement effect [21,22]. In addition, the overall particle size of Au@Ag and its surface plasmon resonance (SPR) could be tuned by adjusting the ratio of Au to Ag to achieve the best enhancement effect for the analyte [23].

The aim of this study was to explore the feasibility of using Au@Ag as SERS substrate to analyze flusilazole in fruits. Au@Ag varied in particle size and the ratio of two metals were tested to achieve the best enhancement effect for detection of flusilazole. This study proposed a SERS-based quick and sensitive analytical method that could be used to detect other pesticides in fruits and vegetables.

## 2. Materials and Methods

### 2.1. Preparation of Standard Solutions

Flusilazole purchased from Sigma-Aldrich (≥99%, Sigma-Aldrich, St. Louis, MO, USA) was dissolved into acetonitrile (HPLC reagent, J&K Scientific, Logan, UT, USA) aqueous solution (50%, *v*/*v*) to prepare flusilazole standard solution with different concentrations. (0.1, 0.2, 0.5, 1 and 2 mg/L). 

### 2.2. Synthesis of Au@Ag Nanoparticles

Au@Ag NPs was prepared by citrate reduction and seed-induced growth methods which described in our previous study [23]. Firstly, gold colloids were obtained as seeds with the Frens method which is a HAuCl_4_-citrate reduction method in 1973 [24]. Next, l-ascorbic acid (0.1 mol/L, 1.20 mL) and as-prepared Au seed (0.4, 0.5, 0.9 mL) were mixed in a 20 mL glass vial and stirred continuously. AgNO_3_ solution (0.01 mol/L, 2.70 mL) was added drop by drop into the above vial to form Au@Ag NPs with keeping stirring. The sizes of Au@Ag NPs could be adjusted by controlling amount of gold colloids (0.4, 0.5 and 0.9 mL) when silver shell was formed and the color of Au@Ag colloid changed from cream to orange as the increase of gold colloids used. The optical characteristics of gold colloids and three different Au@Ag NPs were performed on ultraviolet-visible (UV-Vis) absorbance spectroscopy (UV3000PC, MAPADA Instruments Ltd., Shanghai, China). The sizes and shapes of the Au@Ag NPs were characterized with a transmission electron microscopy (TEM, JEM-2100F, JEOL Ltd., Tokyo, Japan) and each size of Au@Ag NPs was averaged according to 50 Au@Ag particles in TEM images.

### 2.3. Sample Preparation

The method of extracting flusilazole from pears was based on QuEChERS method which was widely used in pesticide residues pre-treatment analysis [25]. In brief, pear samples were homogenized and spiked with various concentrations of fulsilazole (0-control, 0.1, 0.2, 0.5, 2, 5 μg/g). Different spiked pear (10 g) was then vigorously mixed with acetonitrile (20 mL), 3 g NaCl and 3 g MgSO_4_ for 1 min and centrifuged at 4000 rpm for 5 min. Next some sorbents were added into samples in order to cleanup food matrix. 2 mL supernatant was transferred into 15 mL centrifuge tube, added 0.6 g MgSO_4_, 0.2 g PSA sorbent and 0.2 g C_18_ sorbent, then dramatically shook for 1 min, then centrifuged at 4000 rpm for 5 min to eliminate non-targeted compounds such as organic acids, pigment, excess water. The final supernatant was moved to a 5 mL tube for the following SERS analysis, the above sample pretreatment steps were performed with 3 replicates for polluted pear at each concentration.

### 2.4. SERS Measurement

The normal Raman spectrum and SERS spectra of flusilazole standard solutions or pear extracts were obtained by a Nicolet DXR microscopy Raman spectrometer (Thermo Fisher Scientific Inc., Waltham, MA, USA) coupled with a 633 nm He-Ne laser with 6 mW laser power, 20× objective with a slit width of 50 cm^−1^. To acquire SERS spectra, 100 μL Au@Ag NPs were mixed with flusilazole standard solution or pear extract (2:1, *v*/*v*) for 10 s. 5 μL intermixture was pipette onto a neat and tidy glass slide and dried at 50 °C to volatilize the solvent. Ten spectra from different locations on the surface of the substrate were averaged for data analysis. Each experiment was repeated in triplicate.

## 3. Results and Discussion

### 3.1. Spectral Features of Flusilazole

**Figure 1:**
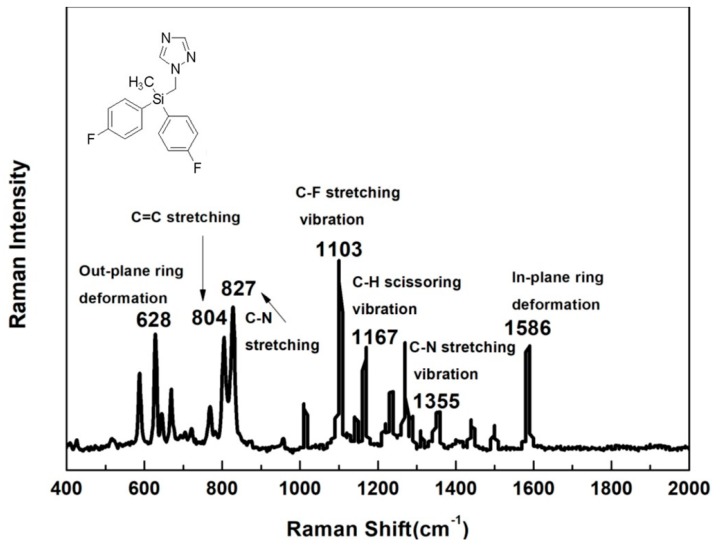
Molecular structure, Raman spectra and the band assignments for characteristic peaks of flusilazole.

Figure 1 shows chemical structure, Raman spectrum and the band assignments for characteristic peaks of flusilazole. The major prominent peaks of flusilazole at 804 and 827 cm^−1^ are attributed to C=C and C–N stretching, respectively [26]. The other major characteristic peaks appeared at 1588 cm^−1^ due to in-plane ring deformation mode of C=C, C–H scissoring vibration (1168 cm^−1^), C–F stretching vibration (1103 cm^−1^), C–N stretching vibration (1355 cm^−1^), as well as out-plane ring deformation (628 cm^−1^) [27,28].

### 3.2. Selection of Different Size Au@Ag Nanoparticles for Fulsilazole

It is well known that the size, degree of aggregation and the shape of the nanosubstrates have a crucial effect on the SERS enhancement effect [29,30]. The enhancing effect of particle size on SERS technology are relatively complicated and affected by many factors and the appropriate nanoparticle size for special application of SERS is often based on extensive experimental data through comparing the result of different sizes of nanoparticles as SERS substrate detecting targeted compound [31,32].

**Figure 2:**
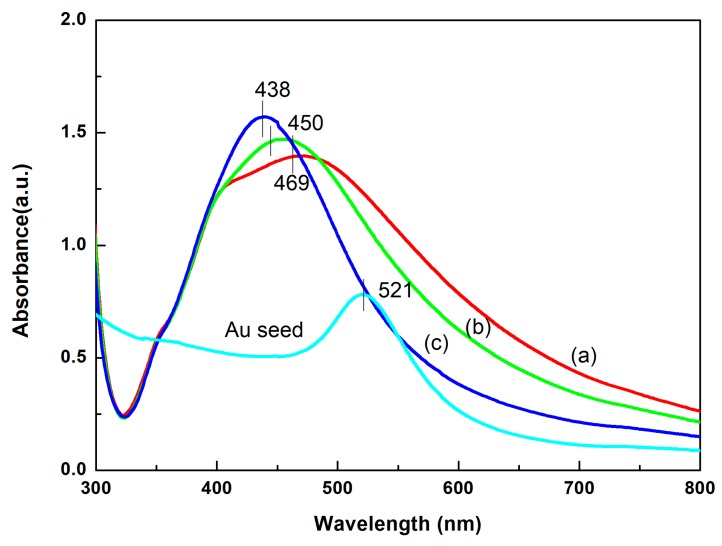
UV-vis spectra of colloidal Au seeds and Au@Ag NPs synthesized with various amounts of Au seeds including (**a**) 0.4 mL; (**b**) 0.5 mL; (**c**) 0.9 mL.

**Figure 3:**
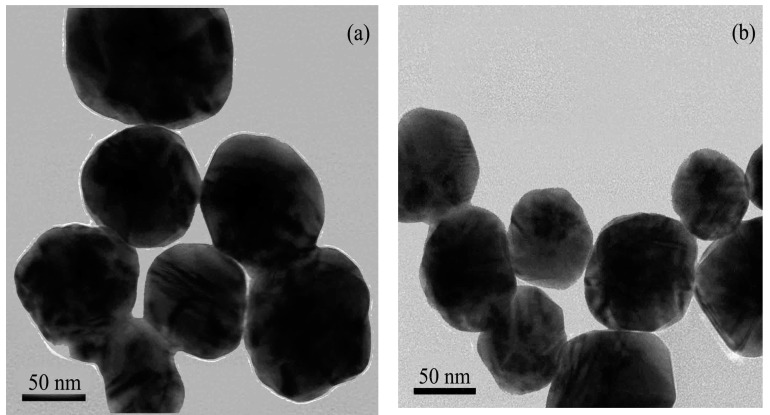

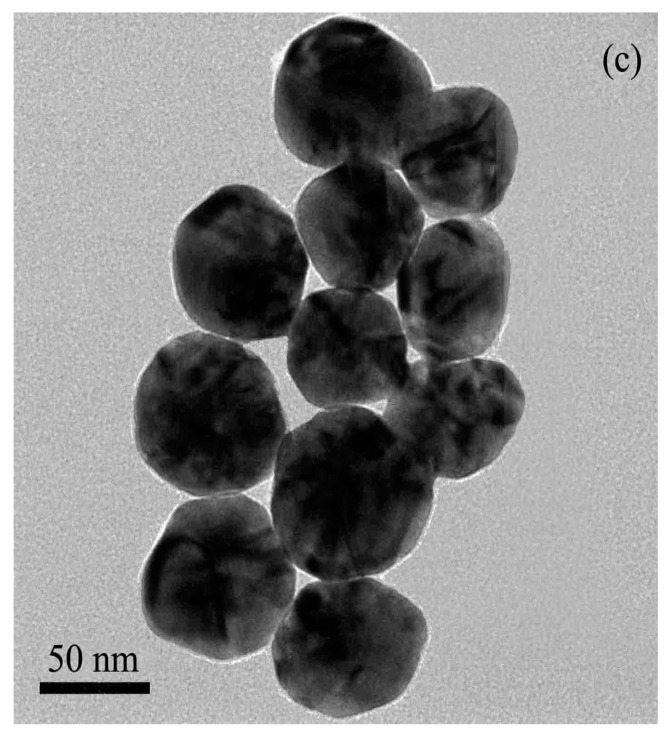
Transmission electron microscopy images of Au@Ag NPs synthesized with different amounts of Au seeds including (**a**) 0.4 mL; (**b**) 0.5 mL and (**c**) 0.9 mL.

Figure 2 shows the surface plasmon resonance (SPR) peaks of gold colloids, which was observed at 521 nm and the SPR peaks of three different Au@Ag NPs were blue-shifted from 469 to 438 nm with the amount of colloidal Au seeds increasing from 0.4 to 0.9 mL. The average diameters of the three substrates were 98 ± 6, 90 ± 7 and 65 ± 4 nm based on the TEM image of Au@Ag NPs (Figure 3). The results indicated the sizes of Au@Ag NPs were decreased with the increase of Au seed amount, which led to the blue-shift of SPR peaks. 

**Figure 4:**
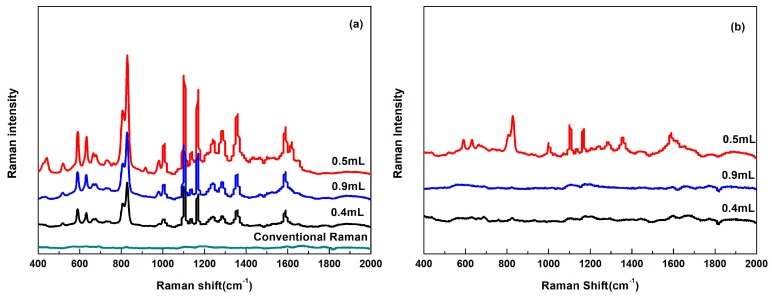
Conventional Raman spectra and surface-enhanced Raman scattering spectra of (**a**) 1 mg/L and (**b**) 0.1 mg/L flusilazole standard solutions using Au@Ag with various amounts of Au seeds including 0.4, 0.5, 0.9 mL.

Compared with SERS spectra of the concentration of 1 and 0.1 mg/L flusilazole, there could not be detected by conventional Raman (Figure 4a), while the SERS intensities of flusilazole were largely affected by the sizes of Au@Ag NPs. As shown in Figure 4a,b, Au@Ag NPs of 90 ± 7 nm led to the best SERS enhancement effect, particularly for 0.1 mg/L concentration. Therefore, the 90 ± 7 nm Au@Ag NPs was used for following experiments for the analysis of flusilazole.

### 3.3. Analysis of Flusilazole Standard Solutions

**Figure 5:**
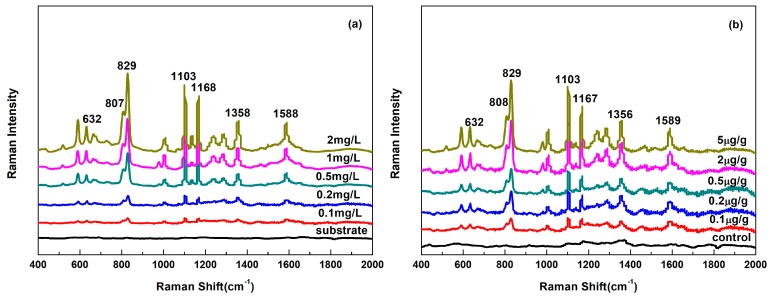
SERS spectra of (**a**) flusilazole standard solutions and (**b**) flusilazole polluted pear extracts.

SERS spectra features of flusilazole standard solutions (Figure 5a) correspond with its conventional Raman spectra (Figure 1), the main characteristic peaks of flusilazole at 632, 807, 829, 1103, 1168, 1358 and 1588 cm^−1^ could be obviously discerned at 0.1 mg/L coupling with the Au@Ag NPs. Compared to that of conventional Raman spectra of flusilazole, there were no clear band shift occurred in the SERS spectrum of flusilazole standard solution but some peaks’ intensities were altered. For instance, the intensity peak of 827 cm^−1^ in the solid Raman spectra was medium, while it became a strong intensity peak in the SERS spectra; on the contrary, 1586 cm^−1^ in the Raman spectrum was a secondary intensity peak, while it became a weak peak in the SERS spectra. The change of relative intensity of characteristic peaks was depended on many factors such as the interaction between targeted chemical molecules and substrate surface, the adsorption sites of substrate, the molecular orientation attached to the substrates and so on [32].

As shown in Figure 5a, the intensity of prominent peaks, such as those at around 632, 807, 829, 1103, 1168, 1358 and 1588 cm^−1^, increased with an increase of flusilazole concentration. A good linear relationship between the intensity of prominent peaks and the flusilazole concentrations was obtained (*R*^2^ = 0.924–0.962), which made it probable to determine flusilazole content with SERS technology (Table 1).

### 3.4. SERS Analysis of Flusilazole in Pears

**Figure 6:**
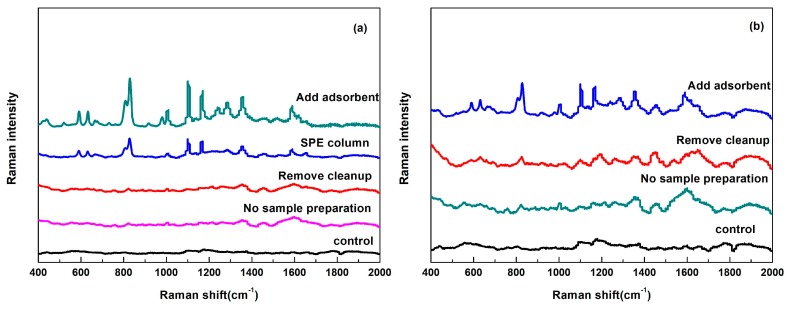
SERS spectra of flusilazole extracts in pears with different sample preparation processes (**a**) 1 μg/g and (**b**) 0.2 μg/g.

Sample pretreatments were needed to decrease the effect of non-targeted chemical components, for example, sugars, organic acids and pigments in pears. The quick, easy, cheap, effective, rugged and safe (QuEChERS) method as a practical sample preparation has been widely used in analysis of residual pesticide residues in fruits and vegetables [33]. This method is divided into two steps: extraction and cleanup. As shown in Figure 6, without sample preparation or removing cleanup, flusilazole cannot be detected, due to sample matrix interference. The sample can be further purified by adding adsorbents or through solid phase extraction (SPE) cartridge, however SPE column is very expensive and both methods are effective (Figure 6a). During the whole steps of QuEChERS method, addition of adsorbents was one of most important steps to remove non-targeted components, for the amounts of adsorbent too little or high could not obtain good SERS enhancement. The amount of each absorber dosage was determined as 0.6 g MgSO_4_, 0.2 g C_18_ and 0.2 g PSA according to several experiments’ results (Table 2). The typical SERS spectra of flusilazole extracts from pears and the major characteristic peaks were consistent with flusilazole standard solutions, which were shown in Figure 5b. There was no other peak appeared in pear extracts, the relative intensity of primary peaks changed comparing with that of standard solutions, which might be due to the interference of pear matrix. 

As shown in Table 3, a good linear relationship between the intensity of prominent peaks and pears extracts was achieved (*R*^2^ = 0.924–0.962). The regressive results of extracts were not as good as those for standard solutions, indicating some non-targeted components in the pear extracts might hinder the adsorption of flusilazole onto the Au@Ag surface and caused less satisfied analysis results for pear extracts. The highest linear relationship was acquired for the peak at 632 cm^−1^, *R*^2^ with value of 0.922, which showed the potential of using SERS for analysis of flusilazole in pears.

## 4. Conclusions

Three different sizes of Au@Ag NPs were easily prepared and used to detect flusilazole standard solutions and in pear extracts. The Au@Ag NPs sizes of 90 ± 7 nm showed the highest enhancement effect, the minimum detectable concentration of flusilazole standard solution and flusilazole in pear extracts were 0.1 mg/L and 0.1 μg/g, respectively. The QuEchERS method, applied to sample preparation, is significant to eliminate or reduce the impact of non-targeted ingredients in food samples for the high sensitivity of SERS technology. SERS can provide a fast, sensitive, economical method to determine flusilazole in pears, which shows the potential for wide applications in residual pesticides in vegetables or fruits. 

## Figures and Tables

**Table 1:** Linear relationship between the different concentrations of flusilazole standard solution (0.1–2 mg/L) and the intensities of prominent characteristic peaks in the surface-enhanced Raman scattering spectra.

-	Peaks/cm^−1^	Regression Equation	*R*^2^
Standard Solution	632	*Y* = 1300.06*x* + 513.57	0.924
-	807	*Y* = 2186.26*x* + 498.59	0.956
-	829	*Y* = 4242.81*x* + 1044.33	0.951
-	1103	*Y* = 3644.11*x* + 783.79	0.952
-	1168	*Y* = 2940.66*x* + 549.86	0.962
-	1358	*Y* = 1677.96*x* + 368.57	0.946
-	1588	*Y* = 1511.46*x* + 564.80	0.924

**Table 2:** Influence of three different amounts of sorbents on SERS detect of flusilazole.

Absorber Dosage			Flusilazole’s Concentration
MgSO_4_	PSA	C_18_	0.1 μg/g
0.45 g	0.1 g	0.1 g	-
0.6 g	0.1 g	0.1 g	-
0.6 g	0.2 g	0.1 g	-
0.6 g	0.2 g	0.2 g	+
0.45 g	0.2 g	0.2 g	-

+: Flusilazole can be detected; -: Flusilazole can not be detected.

**Table 3:** Linear relationship between the intensities of prominent characteristic peaks and the different concentrations of flusilazole in the SERS spectra of pears extracts.

-	Peaks/cm^−1^	Regression Equation	*R*^2^
Pear Extracts	632	*Y* = 232.05*x* + 219.72	0.922
-	808	*Y* = 365.26*x* + 302.72	0.886
-	829	*Y* = 749.85*x* + 533.64	0.914
-	1103	*Y* = 655.32*x* + 557.58	0.921
-	1167	*Y* = 442.63*x* + 525.25	0.879
-	1356	*Y* = 301.85*x* + 450.82	0.741
-	1589	*Y* = 216.17*x* + 350.04	0.708
